# Efficacy and Safety of Anakinra in Colchicine-Resistant or -Intolerant Familial Mediterranean Fever: A Single-Center Real-Life Experience

**DOI:** 10.3390/medicina61050792

**Published:** 2025-04-25

**Authors:** Tuğba Ocak, Havva Nur Köse, Burcu Yağız, Belkıs Nihan Coşkun, Ediz Dalkılıç, Yavuz Pehlivan

**Affiliations:** 1 Department of Rheumatology, Bursa City Hospital, 16250 Bursa, Turkey; 2 Department of Internal Medicine, Faculty of Medicine, Uludag University, 16285 Bursa, Turkey; havvanurkosee@gmail.com; 3 Department of Rheumatology, Faculty of Medicine, Uludag University, 16285 Bursa, Turkey; burcuyilmaz_84@hotmail.com (B.Y.); belkisnihanseniz@hotmail.com (B.N.C.); edizinci@hotmail.com (E.D.); drypehlivan@gmail.com (Y.P.)

**Keywords:** anakinra, colchicine-intolerant, colchicine-resistant, familial mediterranean fever

## Abstract

Familial Mediterranean Fever (FMF) is characterized by recurrent febrile attacks and serositis. While colchicine is the primary treatment for FMF, some patients present resistance or intolerance with respect to this drug. Anakinra—an IL-1 receptor antagonist—has demonstrated efficacy in colchicine-resistant or -intolerant FMF patients. *Background and Objectives:* This study aimed to evaluate the clinical characteristics, treatment duration, response to therapy, dose interval modifications, and long-term outcomes in FMF patients treated with anakinra. *Materials and Methods:* We retrospectively analyzed data from 68 FMF patients who were colchicine-resistant or -intolerant and received anakinra treatment. *Results:* The median patient age was 40.2 years, with a predominance of female patients (57.3%). The median follow-up duration for patients treated with anakinra was 34.2 months. Anakinra dosing was successfully extended in 30.8% of patients. Eight patients discontinued anakinra due to remission, with a median remission duration of 18.4 months. In a subgroup analysis of 57 patients treated with anakinra for at least 12 months, a significant decrease was observed in Pras scores at 0 months, 3 months, and 12 months, as well as in Erythrocyte Sedimentation Rate, C-reactive protein, and Serum Amyloid A values (all *p* < 0.001). Statistically significant decreases in 24 h proteinuria values were found between 0 and 3 months, 3 and 12 months, and 0 and 12 months (*p* = 0.011, *p* = 0.006, and *p* = 0.007, respectively). Anakinra use in pregnancy and kidney transplant recipients was well tolerated. Dose extension and treatment discontinuation in remission are feasible strategies. *Conclusions:* These findings support the use of anakinra as a good treatment option in selected patients.

## 1. Introduction

Familial Mediterranean Fever (FMF) is a monogenic autoinflammatory disease inherited in an autosomal recessive manner [1]. The disease is mainly characterized by periodic episodes of fever, peritonitis, pleuritis, arthritis, and erysipelas-like rashes [2]. Amyloidosis is the most important cause of morbidity and mortality in FMF. Pathogenic mutations in the Mediterranean fever gene (MEFV) lead to activation of the pyrin inflammasome and dysregulated expression of interleukin (IL)-1β [3,4]. Colchicine—a tricyclic-neutral alkaloid—is the main drug used to treat FMF, which prevents attacks and amyloidosis in most FMF patients [5]. However, 5–10% of patients are resistant to colchicine or intolerant of the drug due to gastrointestinal side effects [6]. For these patients, IL-1 inhibitors are the preferred alternative. In our country, the IL-1 inhibitors used for colchicine-resistant or -intolerant FMF patients include anakinra and canakinumab.

Anakinra is a recombinant form of the IL-1 receptor antagonist [7]. In the only randomized controlled trial involving colchicine-resistant FMF patients, the number of attacks was significantly lower in the anakinra group compared to the placebo group [8]. A review of case reports or series treated with anakinra in FMF revealed a complete response in 77% of 64 patients [9]; additionally, a decrease in the frequency of attacks and inflammation was seen in 19% of patients in the same study [9]. Various studies have demonstrated that IL-1 inhibition is a relatively safe and effective alternative for FMF patients who are resistant to or intolerant of colchicine [8,9,10,11,12]. Anakinra is administered as a daily subcutaneous injection of 100 mg, which may make it difficult for patients to adhere to treatment. There is no consensus on extending the dose interval or discontinuing treatment in patients who achieve remission with anakinra [10,11,12]. Moreover, limited studies have examined the long-term follow-up of patients whose treatment was discontinued due to remission [10]. In our study, we aimed to evaluate the clinical characteristics, duration of medication use, response to treatment, and dose interval extending times, for which there are limited data in the literature, as well as the follow-up time in remission in FMF patients treated with anakinra.

## 2. Materials and Methods

### 2.1. Study Population

Our study included patients who were treated with anakinra at the Bursa Uludağ University Faculty of Medicine between 2014 and 2024. All patients were diagnosed with FMF according to the Tel-Hashomer criteria [13]. There were 82 patients for whom the anakinra treatment was initiated with a diagnosis of FMF. Among these patients, 14 patients who did not have regular follow-ups in our center and whose clinical data were missing in the hospital’s electronic system were excluded from the study. Therefore, the number of patients included in the study was 68, all of whom were colchicine-resistant or -intolerant. Colchicine resistance was defined as at least one attack per month despite taking the maximum tolerated dose of colchicine [14], while colchicine intolerance was defined as an inability to increase the effective dose of colchicine due to gastrointestinal side effects, including diarrhea, nausea, and abdominal pain [15].

### 2.2. Study Design and Data Collection

**Figure 1:**
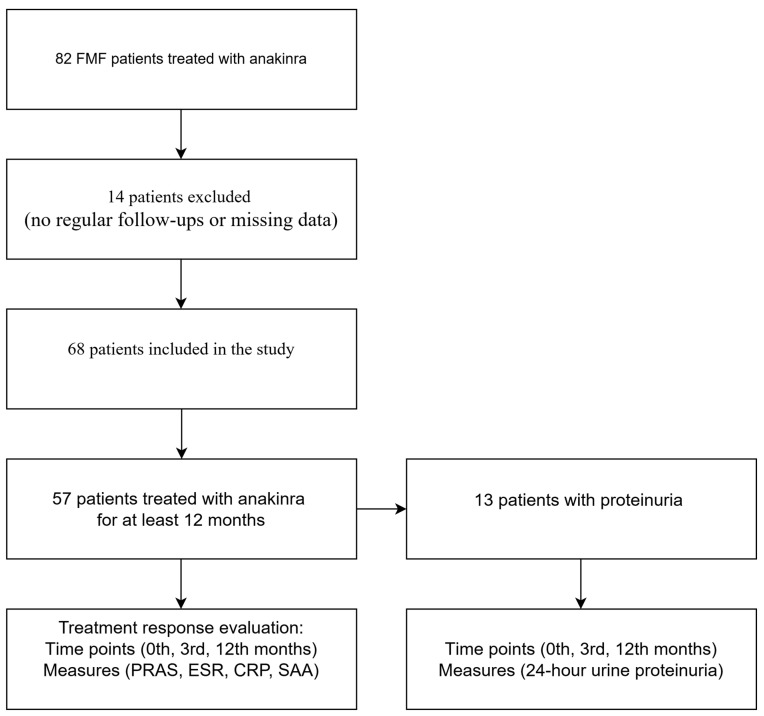
A diagram of the study design. FMF = Familial Mediterranean Fever; PRAS = Disease Severity Score; ESR = Erythrocyte Sedimentation Rate; CRP = C-reactive protein; SAA = Serum Amyloid A.

Demographic characteristics of the patients, attack characteristics at diagnosis, presence of the MEFV gene, presence of amyloidosis, family history of FMF, colchicine doses before the anakinra treatment, duration of disease at initiation of anakinra treatment, duration of follow-up on anakinra treatment, anakinra treatment doses, anakinra treatment dose interval extending times, anakinra use in pregnancy, anakinra use in renal transplant patients, and reasons for treatment discontinuation were retrospectively reviewed using the hospital’s electronic system. The diagnosis of amyloidosis in patients was confirmed through tissue biopsy. A subgroup analysis was performed for 57 patients treated with anakinra for at least 12 months, in order to evaluate the response to treatment with anakinra. Patient responses were assessed at 0 months, 3 months, and 12 months. Pras score, Erythrocyte Sedimentation Rate (ESR), C-reactive protein (CRP), and Serum Amyloid A (SAA) levels were used to assess the response to treatment. In 13 patients with proteinuria treated for at least 12 months, the proteinuria values were also evaluated at the start of treatment, after 3 months, and after 12 months. The Pras score includes age at onset of the disease, frequency of relapses, colchicine dose administered to control relapses, joint involvement, erysipelas-like erythema, and presence of amyloidosis [16]. A study flow chart is displayed in Figure 1. Remission was defined as the absence of relapses in the last 6 months and typical acute phase response values in the relapse-free period. Insufficient response was defined as recurrence of >6 typical FMF attacks per year or >3 typical FMF attacks within 4–6 months, despite receiving the regular dose. Non-compliance was defined as the patient’s reluctance to take the medication due to daily injections.

### 2.3. Statistical Analysis

Statistical analysis was conducted using SPSS (Statistical Package for Social Sciences) version 26.0. Descriptive statistics were obtained for the socio-demographic, clinical, and laboratory parameters of the patients. The normality of variables was assessed using the Shapiro–Wilk and Kolmogorov–Smirnov tests. Quantitative data are expressed as mean ± standard deviation when normally distributed and median (minimum, maximum) when non-normally distributed. The Friedman test was performed to evaluate the response to treatment and determine whether there were any relationships between the Pras score, ESR, CRP, SAA, and 24 h proteinuria levels at months 0, 3, and 12. Then, the Wilcoxon signed-rank test was used for the comparisons between visits at months 0 and 3, 0 and 12, and 3 and 12. Bonferroni correction was performed for three separate paired group comparisons, and a new significant *p*-value < 0.0167 was accepted.

## 3. Results

The demographic and clinical characteristics of 68 patients treated with anakinra for FMF are shown in Table 1. The median age of the patients was 40.2 years (min–max range: 21.8–74.7), and 39 (57.3%) were female. The median time between diagnosis and starting the treatment with anakinra was 8.2 (min–max range: 1.1–23.9) years. The most common symptom at the time of diagnosis was abdominal pain, observed in 61 patients (89.7%). The MEFV mutation was analyzed in 60 patients; among these, 18 were homozygous for M694V, 15 were heterozygous for M694V, 8 were homozygous for V726A, 4 had a combination of M694V/R202Q, 3 were heterozygous for M680I, 3 had the M694V/M680I mutation, 3 had M694V/V726A, 3 had M694V/E148Q, and 1 patient had the M680I/E148Q mutation. Two patients were negative for the MEFV mutation. Amyloidosis was present in 15 patients. Amyloidosis was diagnosed in nine patients by renal biopsy, in two patients by terminal ileum biopsy, in two patients by rectal biopsy, and in two patients by colon biopsy. The most common concomitant disease was hypertension (*n* = 19, 27.9%). All patients were treated with colchicine before the treatment with anakinra, and the median colchicine dose was 2 (min–max range: 1–2) mg. The median follow-up time for patients treated with anakinra was 34.2 (min–max range: 1–118.9) months. There were 37 patients with a follow-up time of more than 30 months, in which Pras scores were evaluated. The median Pras score after 30 months was 4 (1–6). Of the 68 patients, 63 continued colchicine alongside the anakinra treatment. Anakinra was started at 100 mg/day in all patients.

The anakinra dose interval was extended in 21 patients (30.8%) in remission during the treatment. The median time between the start of anakinra treatment and the extension of the anakinra dose interval was 16.6 (min–max range: 2.3–62.2) months. The dose interval extension was initially 100 mg every 2 days. In the follow-up phase, the anakinra dose was continued at 100 mg every 3 days in three patients with extended dose intervals. Over a median follow-up period of 26.5 months (min–max range: 0.9–116.5 months), no disease reactivation requiring an increase in the anakinra dose was observed in patients whose dose intervals were extended. The anakinra treatment was discontinued in eight patients whose dose interval was extended, as they were in remission. The median duration of follow-up in remission after discontinuation of the anakinra treatment was 18.4 (min–max range: 8–47.5) months in patients whose treatment was discontinued due to remission. Disease activation was observed in one of the patients whose treatment was discontinued due to remission after 8 months and in another after 8.4 months, for whom treatment with canakinumab was initiated. Six of the patients whose treatment was discontinued due to remission were followed-up with the colchicine treatment alone.

The demographic and clinical characteristics of the 57 patients treated with anakinra for at least 12 months are shown in Table 2.

### 3.1. Efficacy and Side Effects

Fifty-seven patients were treated with anakinra for at least 12 months. To assess the efficacy of anakinra treatment, the Pras score, ESR, CRP, and SAA levels were evaluated at months 0, 3, and 12. The *p*-values of all parameters were less than 0.001, as shown in Table 3. Between months 0 and 3, the *p*-values of all parameters analyzed for efficacy assessment were less than 0.001. Except for Pras scores at months 3 and 12 (*p* ≤ 0.001), the *p*-values of all parameters analyzed for efficacy assessment were less than 0.001. The *p*-values of all parameters analyzed for efficacy assessment between months 0 and 12 were less than 0.001.

There were statistically low correlations between the CRP and SAA values after 0 months (r = 0.399, *p* = 0.002), as well as after 3 months (r = 0.302, *p* = 0.022). After 1 year, there was no statistical correlation between the CRP and SAA values (*p* = 0.06).

Proteinuria was present in 14 patients at the start of anakinra treatment. In 13 patients who had proteinuria at baseline and were treated for at least 12 months, the median 24 h urine proteinuria was 5895 mg (min–max range: 710–15,059) at month 0, 5144 mg (min–max range: 180–10,868) at month 3, and 900 mg (min–max range: 250–9200) at month 12. There was a significant correlation between the 24 h proteinuria values at the month 0, 3, and 12 visits (*p* < 0.001). Statistically significant decreases in 24 h proteinuria values were found between 0 and 3 months, 3 and 12 months, and 0 and 12 months (*p* = 0.011, *p* = 0.006, and *p* = 0.007, respectively).

The treatment with anakinra was discontinued in 25 patients. The reasons for discontinuation are listed in Table 4, from which it can be seen that the most common reason was that the patients were in remission (*n* = 8). In seven patients, the reason for discontinuation was an insufficient response to treatment. In seven patients, the treatment was discontinued due to side effects. The most common side effect was a reaction at the injection site (*n* = 5).

### 3.2. Pregnancy and Other Conditions

Four patients were treated with anakinra during pregnancy. In two of these patients, the treatment was initiated before pregnancy and continued throughout. In the other two patients, the anakinra treatment was started in the 20th and 24th weeks of pregnancy, respectively. No side effects associated with the anakinra treatment were observed in the mothers or their babies. The anakinra treatment was continued in three of these patients, with follow-up periods of 118.9 months, 44.7 months, and 40.1 months. One patient’s treatment was discontinued after 13.5 months due to her good clinical condition. The characteristics of patients treated with anakinra during pregnancy are shown in Table 5.

Six patients with a history of kidney transplantation were treated with anakinra. The median duration of anakinra treatment in these patients was 61.4 months (min–max range: 12.4–96.3). One patient with a history of kidney transplantation died from COVID-19 pneumonia in the 54th month of treatment. In another patient, the anakinra treatment was discontinued after 12.4 months due to diarrhea. The characteristics of patients with a history of kidney transplantation treated with anakinra are shown in Table 6.

## 4. Discussion

Our study revealed statistically significant decreases in Pras score, ESR, CRP, and SAA values in FMF patients treated with anakinra who were resistant to or intolerant of colchicine. It was observed that the dose interval could be extended, and treatment could be discontinued in patients in remission. A decrease in proteinuria levels was observed during the treatment with anakinra in patients who had proteinuria at the start of treatment. In addition, the efficacy and safety of treatment with anakinra in pregnant women and kidney transplant patients were investigated. Our study has the longest previously published median follow-up time that evaluates the efficacy and safety of anakinra treatment in FMF patients.

Colchicine is the standard treatment for FMF, effectively preventing attacks and suppressing sub-clinical inflammation [17,18]. However, a subset of FMF patients either fail to respond to colchicine or develop intolerance of the drug [19]. In our study, 92.6% of the considered patients were colchicine-resistant, while 7.4% were colchicine-intolerant. Similarly, in the study by Marko et al., most patients were resistant to colchicine [11]. Recent studies have demonstrated that IL-1 antagonists can be an effective alternative for FMF patients who are resistant to or intolerant of colchicine. Ben-Zvi et al. showed that the treatment with anakinra was effective and safe in 12 colchicine-resistant FMF patients in a randomized, controlled, double-masked study [8]. Köhler et al. have reported, in a study of 29 FMF patients, that disease symptoms and inflammatory markers were rapidly and permanently suppressed [20]. In a study conducted by Marko et al. with 44 FMF patients, decreases in attacks and inflammatory markers were observed [11]. In a study by Ataş et al., involving 88 FMF patients receiving anakinra, disease activity was improved at the last visit [12]. In our study, similar to other studies, a significant decrease in clinical and inflammatory markers was observed during the treatment with anakinra, compared to the start of treatment. ESR and CRP have been evaluated in previous studies [10,11,12,20,21], among other inflammatory markers. Another important marker used in the monitoring of FMF patients is SAA, an apolipoprotein that is one of the major acute-phase proteins synthesized by the liver [22]. SAA is the precursor of the amyloid fibrils deposited in tissues via AA amyloidosis in untreated FMF patients [23,24,25]. In our study, in contrast to previous studies, SAA levels were determined in addition to ESR and CRP. SAA is not a routine test in Turkey and some other countries. In these centers, CRP can be used to monitor inflammatory activity in FMF [26]. However, in the study conducted by Berkun et al., it was shown that SAA can be high in some FMF patients with normal CRP levels [27]. When the role of SAA in diagnostic and treatment decisions was evaluated, it was concluded that a normal CRP level is not critical for treatment decisions [27]. Therefore, in centers where SAA can be checked, it may be useful to determine the treatment. However, in centers where SAA cannot be checked, CRP can be used as an inflammatory marker for follow-up.

The treatment with anakinra could be a good option to achieve a clinical and anti-inflammatory effect in patients who are resistant to or intolerant of colchicine. When the follow-up time of anakinra was evaluated in previous studies, the median follow-up time was 16.01 months in the study by Uğurlu et al. [10], 16.2 months in the study by Sargın et al. [28], and 18 months in the study by Marko et al. [11]. The median follow-up time was 30 months in the study by Ataş et al. To the best of our knowledge, our study had the longest follow-up time, with a median of 34.2 months.

Anakinra (100 mg/day) is administered subcutaneously. The daily subcutaneous injection route may make compliance with treatment more difficult. It is, therefore, essential to extend the administration interval during follow-up and to be able to discontinue the drug in patients in remission. Data in the literature report that the anakinra dose, dose intervals, and duration of treatment in FMF patients are limited. A few studies have provided information on the administered doses of anakinra [10,11]. However, these studies did not provide information on the transition of duration to doses below 100 mg/day [10,11]. In our study, the dose interval could be extended in 21 patients. The median time between the start of anakinra treatment and the extension of the anakinra dose range was 16.6 (2.3–62.2) months. The duration of treatment continuation with these doses is also essential. In our study, during a median follow-up period of 26.5 (0.9–116.5) months, no activation requiring an increase in drug dose was observed in patients whose anakinra dose interval was extended. This demonstrates that the dose interval can be extended in patients without attacks and sub-clinical inflammation during the follow-up period. Extending the dose interval may improve patient compliance with the drug. In eight patients whose dose interval was extended, the treatment with anakinra was discontinued as the disease was in remission. The abrupt discontinuation of anakinra may trigger a self-repeating inflammatory cycle, due to its short duration of action. Therefore, the treatment with anakinra was discontinued by extending the dose interval to every 2 days, and then to every 3 days. The duration of remission in patients whose treatment was discontinued is also essential. Only one study in the literature has examined this question [10]. In the study by Uğurlu et al., the treatment with anakinra was discontinued in eight patients due to remission [10], and the median duration of remission was 30 months. Three patients who discontinued the anakinra treatment experienced an exacerbation at the end of a median period of 28.7 months, and the other five patients remained in remission with the colchicine treatment [10]. In our study, the median duration of remission was 18.4 (8–47.5) months. In one of our patients, the disease worsened in the eighth month and, in another, a similar observation was made after 8.4 months. Six patients were in remission with the colchicine treatment alone. In patients who were in remission with anakinra, the treatment can be discontinued by gradually extending the dose interval. However, these patients should be closely monitored for disease worsening.

Proteinuria is a complication observed particularly in amyloidosis patients with FMF. In two previous studies, proteinuria was decreased with anakinra [12,21]. In our research, proteinuria decreased during the treatment with anakinra. The decrease in proteinuria suggests that anakinra may prevent complications and systemic inflammation in FMF patients. The most lethal complication of FMF is AA-type amyloidosis, and FMF treatment approaches particularly aim to prevent the development of amyloidosis and amyloidosis-related damage. The role of colchicine in the prevention of amyloidosis has been established [5]; however, its role in the development of amyloidosis remains unclear. Observational studies have suggested that IL-1 inhibition may be effective in FMF patients with AA amyloidosis, and may improve proteinuria [29,30,31]. These studies included short follow-up periods. Anakinra is a newer agent, and the elucidation of its role in reducing proteinuria and preventing amyloidosis requires further randomized controlled trials. Unless there is a contraindication to taking colchicine, the treatment with anakinra should be continued together with colchicine.

Anakinra is classified as pregnancy category B by the Food and Drug Administration (FDA). Data have shown that anakinra is safe for the mother and fetus [32,33]. In one series, 21 of 23 pregnancies treated with anakinra resulted in a healthy birth and one miscarriage. One baby was born with unilateral renal agenesis and an ectopic neurohypophysis [34]. This study’s patient population was mixed and consisted mainly of patients with cryopyrin-associated periodic syndromes [34]. Only three patients had FMF [34]. None of the mothers or infants had an infection [34]. In our study, four patients received the anakinra treatment during pregnancy, and no maternal or infant complications were observed. Due to the increased risk of infection during pregnancy and possible pregnancy complications, anakinra could be a good option for FMF patients who are resistant to or intolerant of colchicine, due to its short half-life.

Increased mortality, morbidity, and chronic graft rejection have been observed in FMF amyloidosis patients with renal transplantation, compared to non-FMF amyloidosis patients [35]. In previous studies conducted in renal transplant patients, no serious adverse effects or drug interactions were observed [21,33]. In our research, one kidney transplant patient died due to COVID-19 pneumonia. As the patient was receiving additional immunosuppressive treatment, it may not be appropriate to define anakinra as the leading cause of this death. In one patient, the treatment with anakinra was discontinued due to diarrhea. No serious infections, side effects, or drug interactions were observed in other kidney transplant patients. Immunosuppressive therapies are used to prevent organ rejection in kidney transplant patients. Therefore, the short half-life of anakinra in FMF patients with kidney transplantation could offer a safety-related advantage in the case of infection.

Safety concerns regarding a medication may limit its use. Most of the adverse reactions reported for anakinra are injection site reactions, which may require topical corticosteroids or systemic antihistamines and usually resolve within one month [36]. Injection site reactions can sometimes lead to discontinuation of anakinra treatment. In our study, the most common side effect was an injection site reaction, and treatment was discontinued in five patients for this reason. No tuberculosis or malignancy was detected during the anakinra treatment in our study.

### Limitations and Strengths

Our study had limitations, such as the facts that it was a single-center study, only a Turkish population was included, it was a retrospective study, and the number of patients was small. Another notable limitation of our study is the lack of a control group. This limits the ability to generalize the results to larger patient populations or other treatment settings. The strengths of our study are that the patients were followed over a long period of time, the dose interval for patients in remission and the duration of anakinra discontinuation were reported, and the follow-up period after anakinra discontinuation was reported. This may be useful for clinicians when making treatment decisions in similar populations.

## 5. Conclusions

Our study demonstrated that anakinra is an effective and safe treatment for colchicine-resistant or -intolerant FMF patients. Significant improvements in inflammatory markers and proteinuria were observed during the treatment with anakinra. In patients in remission, the dose intervals can be extended and further treatment without anakinra is possible. However, the standard treatment approaches for extending the dose intervals and interrupting treatment should be determined through prospective studies with larger numbers of patients.

## Figures and Tables

**Table 1:** Demographic and clinical features of FMF patients treated with anakinra (*n* = 68).

Age (years)	40.2 (21.8–74.7)
Gender (F/M)	39/29
Age at FMF diagnosis (years)	28.1 ± 15
Age at onset of anakinra therapy (years)	36.3 (19.7–71.1)
Indications of anakinra therapy	
Colchicine resistance, *n* (%)	63 (92.6)
Colchicine intolerance/side effects, *n* (%)	5 (7.4)
Time between diagnosis and anakinra therapy (years)	8.2 (1.1–23.9)
Family history of FMF, *n* (%)	36 (52.9)
Fever, *n* (%)	40 (58.8)
Abdominal pain, *n* (%)	61 (89.7)
Chest pain, *n* (%)	10 (14.7)
Arthralgia/Arthritis, *n* (%)	44 (64.7)
Myalgia, *n* (%)	9 (13.2)
Prolonged febrile myalgia, *n* (%)	1 (1.5)
Erysipelas-like erythema, *n* (%)	3 (4.4)
Hepatomegaly, *n* (%)	16 (23.5)
Splenomegaly, *n* (%)	16 (23.5)
Amyloidosis, *n* (%)	15 (22.1)
Comorbidities, *n* (%)	46 (67.6)
Diabetes mellitus, *n* (%)	7 (10.3)
Hypertension, *n* (%)	19 (27.9)
Hyperlipidemia, *n* (%)	18 (26.5)
Pulmonary disease, *n* (%)	14 (20.6)
Chronic renal failure, *n* (%)	11 (16.2)
Coronary artery disease, *n* (%)	8 (11.8)
Spondyloarthropathies, *n* (%)	5 (7.8)
Colchicine dose before anakinra (mg)	2 (1–2)
Duration of anakinra, median (min–max range), (months)	34.2 (1–118.9)

FMF = Familial Mediterranean Fever; F = female; M = male.

**Table 2:** Demographic and clinical features of FMF patients treated with anakinra for at least 12 months (*n* = 57).

Age, years	40.4 (21.8–74.7)
Gender (F/M)	31/26
Age at FMF diagnosis (years)	28 ± 14.9
Age at onset of anakinra therapy (years)	37.9 (19.7–70.3)
Indications of anakinra therapy	
Colchicine resistance, *n* (%)	54 (94.7)
Colchicine intolerance/side effects, *n* (%)	3 (5.3)
Time between diagnosis and anakinra therapy (years)	9.9 (1.1–23.9)
Family history of FMF, *n* (%)	32 (56.1)
Fever, *n* (%)	34 (59.6)
Abdominal pain, *n* (%)	52 (91.2)
Chest pain, *n* (%)	9 (15.8)
Arthralgia/Arthritis, *n* (%)	36 (63.2)
Myalgia, *n* (%)	9 (15.8)
Prolonged febrile myalgia, *n* (%)	1 (1.8)
Erysipelas-like erythema, *n* (%)	1 (1.8)
Hepatomegaly, *n* (%)	15 (26.3)
Splenomegaly, *n* (%)	16 (28.1)
Amyloidosis, *n* (%)	13 (22.8)
Comorbidities, *n* (%)	37 (64.9)
Diabetes mellitus, *n* (%)	5 (8.8)
Hypertension, *n* (%)	15 (26.3)
Hyperlipidemia, *n* (%)	13 (22.8)
Pulmonary disease, *n* (%)	10 (17.5)
Chronic renal failure, *n* (%)	10 (17.5)
Coronary artery disease, *n* (%)	6 (10.5)
Spondyloarthropathies, *n* (%)	4 (7)
Colchicine dose before anakinra (mg)	2 (1–2)
Duration of anakinra, median (min–max range), (months)	39.5 (12.2–118.9)

FMF = Familial Mediterranean Fever; F = female; M = male.

**Table 3:** Comparison of anakinra treatment responses and proteinuria at months 0, 3, and 12.

	Month 0	Month 3	Month 12	*p*	0th–3rd Month *p*	3rd–12th Month *p*	0th–12th Month *p*
PRAS (*n* = 57)	7 (2–11)	4 (1–8)	4 (1–7)	**<0.001**	**<0.001**	**≤** **0.001**	**<0.001**
ESR (mm/h) (*n* = 57)	25 (4–84)	9 (2–68)	9 (2–61)	**<0.001**	**<0.001**	**<0.001**	**<0.001**
CRP (mg/L) (*n* = 57)	20 (2–124)	6 (2–32)	3 (2–21)	**<0.001**	**<0.001**	**<0.001**	**<0.001**
SAA (mg/L) (*n* = 57)	81.7 (3–390)	18 (3–150)	6 (2.9–139)	**<0.001**	**<0.001**	**<0.001**	**<0.001**
24 h urine proteinuria values (mg) (*n* = 13)	5895 (710–15,059)	5144 (180–10,868)	900 (250–9200)	**<0.001**	**0.011**	**0.006**	**0.007**

PRAS = Disease Severity Score; ESR = Erythrocyte Sedimentation Rate; CRP = C-reactive protein; SAA = Serum Amyloid A. Statistically significant values are shown in bold.

**Table 4:** Reasons for anakinra treatment discontinuation (*n* = 25).

Remission	8
Insufficient response	7
Non-compliance	1
Pregnancy plan	1
Exitus	1
Side effects	7
Skin reaction	5
Diarrhea	1
Leukopenia	1

**Table 5:** Characteristics of patients treated with anakinra during pregnancy (*n* = 4).

	Patient 1	Patient 2	Patient 3	Patient 4
Age at FMF diagnosis (years)	8.2	23.8	17.4	33.3
Age at onset of anakinra therapy (years)	28.5	35	28	34.6
Duration of anakinra (months)	44.7	118.9	13.5	40.1
0th month PRAS	8	8	9	5
0th month ESR (mm/h)	51	29	28	23
0th month CRP (mg/L)	124	37	15.6	2
0th month SAA (mg/L)	272	178	81.7	87
3rd month PRAS	6	5	5	3
3rd month ESR (mm/h)	38	22	17	12
3rd month CRP (mg/L)	22	12	4	2
3rd month SAA (mg/L)	48	38	23	24
12th month PRAS	6	5	4	3
12th month ESR (mm/h)	24	28	9	2
12th month CRP (mg/L)	5	7	2	2
12th month SAA (mg/L)	41	14	3	6

FMF = Familial Mediterranean Fever; PRAS = Disease Severity Score; ESR = Erythrocyte Sedimentation Rate; CRP = C-reactive protein; SAA = Serum Amyloid A.

**Table 6:** Characteristics of patients with a history of kidney transplantation treated with anakinra (*n* = 6).

	Patient 1	Patient 2	Patient 3	Patient 4	Patient 5	Patient 6
Gender	M	F	M	F	M	M
Age at FMF diagnosis (years)	31.7	39.7	23.2	55.4	20.2	20.7
Age at onset of anakinra therapy (years)	53	41.3	39.6	57.8	26.4	42.9
Duration of anakinra (months)	68.8	82.2	54	96.3	45.1	12.4
0th month PRAS	5	4	9	9	8	10
0th month ESR (mm/h)	58	33	56	65	20	32
0th month CRP (mg/L)	25	9.9	7.1	3	10.1	3.5
0th month SAA (mg/L)	50	13	30	124	6.2	82
3rd month PRAS	5	2	5	7	7	8
3rd month ESR (mm/h)	24	14	20	23	12	24
3rd month CRP (mg/L)	20	9	5	3	4	3
3rd month SAA (mg/L)	40	8	23	8	4	12
12th month PRAS	5	1	5	7	7	6
12th month ESR (mm/h)	4	7	4	25	4	18
12th month CRP (mg/L)	21	5.6	4	3.1	2	2
12th month SAA (mg/L)	25	4	12	4	3	4.8

F = female; M = male; FMF = Familial Mediterranean Fever; PRAS = Disease Severity Score; ESR = Erythrocyte Sedimentation Rate; CRP = C-reactive protein; SAA = Serum Amyloid A.

## Data Availability

The data presented in this study are available on request from the corresponding author.
